# Crystallisation and Microstructure of Sludge Particles in AlSi7Mg Secondary Alloys with Increased Iron Content

**DOI:** 10.3390/ma18214921

**Published:** 2025-10-28

**Authors:** Jarosław Piątkowski, Stanisław Roskosz, Sebastian Stach, Marcin Górny

**Affiliations:** 1 Faculty of Materials Engineering, Silesian University of Technology, Krasińskiego 8 Street, 40-019 Katowice, Poland; jaroslaw.piatkowski@polsl.pl; 2 Faculty of Science and Technology, Institute of Biomedical Engineering, University of Silesia in Katowice, Będzińska 39 Street, 41-205 Sosnowiec, Poland; sebastian.stach@us.edu.pl; 3 Faculty of Foundry Engineering, AGH University of Krakow, Reymonta 23 Street, 30-059 Krakow, Poland; mgorny@agh.edu.pl

**Keywords:** high-pressure die-casting, Al-Si-Mg alloys, sludge particles, crystallisation, differential scanning calorimetry, phase transformations

## Abstract

The significant increase in the importance of silumin recycling in the context of sustainable development is driven by tangible ecological and economic benefits. However, the primary technological challenge associated with using scrap is the accumulation of iron, which promotes the formation of undesirable sludge particles, degrading the alloy’s mechanical properties. This paper presents a description of the phase transformations in an AlSi7Mg alloy with increased iron and manganese content. Analysis of data from Differential Scanning Calorimetry (DSC) revealed the primary crystallisation of sludge particles (SP) and the pre-eutectic precipitation of the α-Al_15_(Fe,Mn)_3_Si_2_ phase, which replaced the β-Al_5_FeSi phase. The remaining constituents of the AlSi7Mg alloy structure—α(Al) solid solution dendrites, the α(Al)+β(Si) eutectic, and the Mg_2_Si phase—crystallise regardless of the iron, manganese, and chromium content. It was established that the increase in the crystallisation temperature of SP, rich mainly in the elements mentioned above, is directly proportional to the increase in the value of the sludge factor (SF) and ranges from 620 °C (for SF~1.3%) to approx. 645 °C (for SF~3.1%). SEM studies revealed that the combined increase in iron and manganese content not only raises the precipitation temperature of SP but also alters its morphology from single polyhedra to compact, “cluster-like” structures. To avoid the presence of sludge particles in the AlSi7Mg alloy, which have an unfavourable morphology and reduce the yield of the melting process, the SF for high-pressure die-casting should not exceed 2.0%.

## 1. Introduction

Due to their high strength-to-weight ratio, good castability, corrosion resistance, and recyclability, foundry aluminium alloys are frequently used in many industrial sectors, especially in the broad field of transport [1,2]. Their most common and detrimental impurity is iron [3,4]. Its impact on mechanical properties, particularly plasticity, is especially harmful when the iron content exceeds approx. 0.4 wt.% (for gravity castings) [5] and approx. 1.5 wt.% (for high-pressure die-castings) [6,7]. This occurs because iron has low solubility in the α(Al) solid solution (from 0.052% at 655 °C to 0.0051% at 450 °C) [8]. Consequently, it exhibits a strong tendency to combine with other elements, forming intermetallic phases with different stoichiometries, morphologies, and sizes [9,10]. The most undesirable phases are mainly β-Al_5_FeSi (β-Fe) and α_H_-Al_8_Fe_2_Si [11]. In Al-Si-Mg alloys with a content of 0.8 to 1.2 wt.% Mg, besides the Mg_2_Si phase, there is a high probability of crystallisation of the Al_9_Fe_2_Si phase with a monoclinic structure and the hexagonal Al_8_FeMg_3_Si_2_ phase. Additionally, metastable ternary phases β-Al_4_FeSi (25.4 wt.% Fe and 25.5 wt.% Si) and β-Al_3_FeSi (33.9 wt.% Fe and 16.9 wt.% Si) crystallise under conditions of thermodynamic non-equilibrium [8]. In Al-Si-Mg alloys with copper additions, Al_2_Cu, Al_2_CuMg, and Al_6_CuMg_4_ phases, which are in equilibrium with the α(Al) solid solution [12], as well as Al_6_FeCu and Al_7_FeCu_2_ phases [13], can crystallise.

Regardless of the chemical composition of aluminium alloys, the most unfavourable phase morphologically is β-Al_5_FeSi [14,15]. Its low coherence with the matrix and large size increase the brittleness of the alloy, complicate the machining of castings, and reduce tensile strength [14,16]. Furthermore, the plate-like/needle-like morphology of the β-Fe phase impairs the castability, ductility, and corrosion resistance of aluminium alloys. Moreover, according to studies [17,18], a large proportion of β-Fe phases contributes to an increase in shrinkage porosity, regardless of whether the porosity results from the presence of oxide films (termed “bifilms” by Campbell) [19,20] in Al-Si alloys or from the presence of β-Fe phases [5,21].

Since iron cannot be removed from liquid silumin [1], several methods have been developed to neutralise its detrimental effect on the service properties of Al-Si alloys. Examples include dilution with pure aluminium, sedimentation and filtration [22], treatment with an electromagnetic field [23], and other methods [24,25]. There are also solutions involving the introduction of so-called “chemical correctors” into liquid Al-Si(Fe) alloys, which change the unfavourable morphology of the β-Fe phase. Such additives can be strontium and phosphorus [26,27], as well as manganese, molybdenum, cobalt, and chromium [1]. Due to the similarity of the crystal lattice and lattice parameters to those of iron, these elements alter the morphology of the β-Fe phase from plate-like to so-called “Chinese script” or polyhedral blocks, thereby increasing their precipitation and making them smaller [28,29]. The best-described and most popular alloying addition is manganese [7,10], although some research indicates that chromium can be equally effective [30,31]. These additives cause the transformation of β-Al_5_FeSi phases into α-Al_X_(Fe,Mn,Cr)_Y_Si_Z_-type phases (x = 12; 15; 19; 20; y = 3; 5; z = 1; 2; 3) [1], of which Al_15_(Fe,Mn)_3_Si_2_ or Al_12_(Fe,Mn)_3_Si_2_ are the most frequently formed. The Al_19_Fe_4_MnSi_2_ phase, considered isomorphic with Al_20_Fe_5_Si_2_, can also crystallise [32]. In these phases, iron, manganese, and chromium atoms can substitute for one another in the same body-centred cubic (bcc) crystal structure [33]. As reported by studies [14,34], the morphology and size of α-Al_X_(Fe,Mn,Cr)_Y_Si_Z_-type phases vary and depend on several factors, including chemical composition, cooling rate (i.e., the degree of alloy undercooling, ΔT), and the type of technology.

However, the introduction of “chemical correctors” for the β-Fe phase, especially in an improper ratio to the iron content (recommended ratio Mn/Fe < 1), leads to the formation of undesirable sludge particles. This sediment most often consists of aluminium and magnesium oxides [10] and multi-component phases rich in iron along with aluminium, silicon, manganese, and chromium [30,35]. Since sludge has a higher density than liquid silumin, it settles at the bottom of the furnace (ladle, crucible), reducing the efficiency of the casting process. The worst scenario occurs when, for example, sediment particles enter the liquid alloy during the transfer of molten metal from the furnace to the ladle, altering its chemical composition. Then, “entrapped” in the casting, they form so-called “hard spots,” which reduce the mechanical properties, especially the ductility and castability of the alloy, and complicate the machining of castings. Furthermore, the formation of sludge particles causes a local depletion of the alloy in iron, manganese, and chromium, which increases the tendency of Al-Si alloys to hot tear onto dies [36]. For this reason, the presence of sludge particles is hazardous during high-pressure die casting, where a turbulent flow of the liquid alloy occurs. To avoid this, chemical composition limits for the sludge, defined by the so-called “sludge factor” (SF), are known [6,31]. According to [36,37], the SF should be from approx. 1.2 to 1.9%, although sometimes this range is even wider, from 0.6 to 2.85% [6]. The morphology of sludge particles depends mainly on the heat extraction rate, the chemical composition of the alloy, and especially on their crystallisation, which is why determining the precipitation temperature of sludge particles is very important, as shown by research [37,38]. However, this determination is challenging, complex, and dependent on numerous factors.

Consequently, despite numerous studies on the formation of sediment sludge and the role of sludge particles in Al-Si alloys, some aspects still require clarification. This primarily concerns the description of the crystallisation of sludge particles in Al-Si-Mn-Cr alloys with an increased iron content, as the main impurity in secondary materials, whose growing importance in the processing of aluminium alloys is evident and results from various ecological and economic aspects [39,40].

## 2. Materials and Methods

### 2.1. Aim and Scope of Research

**Figure 1:**
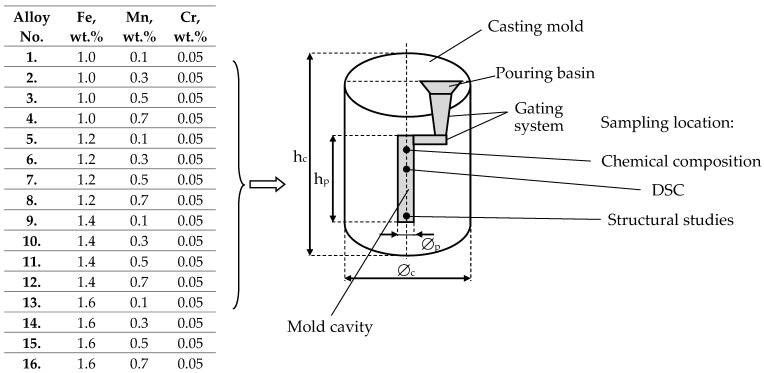
Research plan for the AlSi7Mg alloy with varying iron and manganese content and the location of sample collection for testing; h_c_—height of the casting mould: 160 mm, h_p_—height of the cast sample: 80 mm, ϕ_p_—diameter of the casting mould: 60 mm, ϕ_c_—diameter of the cast sample: 25 mm.

The research aims to characterise the crystallisation process of sludge particles in a high-pressure die-cast AlSi7Mg alloy with increased iron content and additions of manganese and chromium. To achieve the adopted goal, the scope of the research includes, among others:

- performing melts of the AlSi7Mg alloy with varying shares of iron, manganese, and chromium according to the experimental plan (Figure 1),
- analysis of phase transformations occurring during heating and cooling using Differential Scanning Calorimetry (DSC) and determination of the range of characteristic temperatures of these transformations,
- determination of the heat of reaction (enthalpy ΔH) for AlFeMn phases and sludge particles,
- metallographic examinations.

An AlSi7Mg alloy with increased iron content was selected for the study. The choice of alloy was dictated by its good weldability, high corrosion resistance, and good mechanical properties among other Al-Si alloys. This results in its broad application, including various types of castings produced under pressure used in many industrial sectors [1,41], primarily in transportation, such as for cast automobile wheels and for 3D-printed parts with complex shapes [2].

### 2.2. Method of Melting Alloys

The alloy was melted from the following components and master alloys:

- aluminium grade A00 (99.9 wt.% Al),
- technical silicon with a purity of 99.6 wt.% Si,
- AlMg10 alloy (approx. 10 wt.% Mg),
- AlFe25 master alloy (approx. 25 wt.% Fe),
- AlMn50 master alloy (approx. 50 wt.% Mn), as a manganese carrier,
- AlCr10 master alloy (approx. 10 wt.% Cr), as a chromium carrier (Mn and Cr additions were introduced to change the unfavourable morphology of the β-Fe phase).

The charge materials were melted in a Balzers VSG02 induction furnace (Balzers & Co. GmbH, Bad Schönborn, Germany) in a SiC crucible with a capacity of 0.8 L. After melting the components and introducing the manganese and chromium additions (according to studies [6,31], the Mn/Fe ratio was up to 1.0, and Cr/Fe up to 0.3 [30]), the alloys were modified with an AlSr10 master alloy (0.05 wt.%) and refined with Rafglin-3 preparation (Pedmo, Tychy, Poland) in an amount of 0.2 wt.% of the alloy mass. To demonstrate only the influence of manganese, the chromium content was constant and amounted to 0.05 wt.% of the charge mass. To avoid sedimentation and ensure the homogeneity of the metallic bath, the melt was stirred regularly. Upon reaching a temperature of approx. 700 °C and taking a sample for preliminary chemical composition checking, the alloys were cast into a metal mould with a capacity of approx. 400 cm^3^ and a heat extraction rate (from T_liq._ to T_sol._) of approx. 80–100 °C·s^−1^. Chemical composition measurements were performed on the top surface of the ingots, and samples for DSC calorimetric and metallographic examinations were taken from the central part of the cross-section.

### 2.3. Research on Structure, Phase Composition, and Phase Transformation Temperatures

Chemical composition analysis was performed using an optical emission spectrometer for quantitative elemental analysis of solid metal samples, the Foundry-Master Compact 01L00113 from Worldwide Analytical System, featuring CCD technology (SpectroLab, Kleve, Germany). For each sample, 10 measurements were performed, from which the arithmetic mean was calculated.

Investigations of phase transformations using Differential Scanning Calorimetry (DSC) were conducted on a Multi HTC Setaram high-temperature calorimeter (Mettler Toledo, Warszawa, Poland) in a 99.999% Ar (N 50) atmosphere within the temperature range of 450 to 700 °C. Cylindrical samples (weighing approx. 95 mg) were placed in an Al_2_O_3_ measurement crucible. The calorimeter was coupled with the SetSoft computer programme 1.0. Temperature (T) measurement was performed using a Pt-Rh10Pt thermocouple (Wika, Włocławek, Polska). High-purity Al_2_O_3_ powder was used as the reference substance. Before each measurement, the calorimeter was calibrated at a heating and cooling rate of 20 °C min^−1^ (according to the ASTM E1269 standard for determining specific heat by differential scanning calorimetry). For the AlMnFe phase and sludge particles, the area under the peak was chosen, which is proportional to the heat of reaction (enthalpy ΔH) associated with the transformation. Positive ΔH values result from the exothermic reaction of solid phase formation during solidification, and negative ones concern the enthalpy absorbed during heating of the alloy. Repeated DSC tests showed that the temperature difference during heating and cooling was ±1 °C.

Microstructure observations (Light Microscopy-LM) were performed on an Olympus GX71MeF2 light microscope (Olympus Global EMC Ltd., Tipton, UK). SEM studies were performed on a Hitachi S-3400N scanning electron microscope (Hitachi High-Technologies Tokyo, Japan), equipped with a Thermo Noran energy-dispersive X-ray spectrometer (EDS, Thermo Fisher Scientific, Waltham, MA, USA) and a Thermo MagnaRay wavelength-dispersive spectrometer (WDS), and an INCA HKL Nordys II detector for electron backscatter diffraction (EBSD) studies. Ten images were taken, of which the presented images are representative of the microstructure of the tested alloy. Samples for metallographic examination of the sediment sludge were taken from the bottom of the SiC crucible after the alloy solidified. Image acquisition of the structure and the recording process of the AlSi7Mg alloy components after the addition of iron, manganese, and chromium was performed using the AnalySis programme 12.2 using the bright-field observation method.

## 3. Results

### 3.1. Chemical Composition Test Results

The results of the chemical composition tests of the AlSi7Mg alloy with an increasing share of iron and the addition of manganese are presented in Table 1. The sludge factor was determined from the relationship: SF = (1·wt.% Fe) + (2·wt.% Mn) + (3·wt.% Cr) [6].

From the data presented in Table 1, it follows that the silicon content is approx. 7 wt.%, magnesium approx. 0.6 wt.%, and the assumed amounts of manganese and chromium. The remaining elements of the AlSi7Mg alloy also fall within the range given in the standard [42].

### 3.2. DSC Test Results

**Figure 2:**
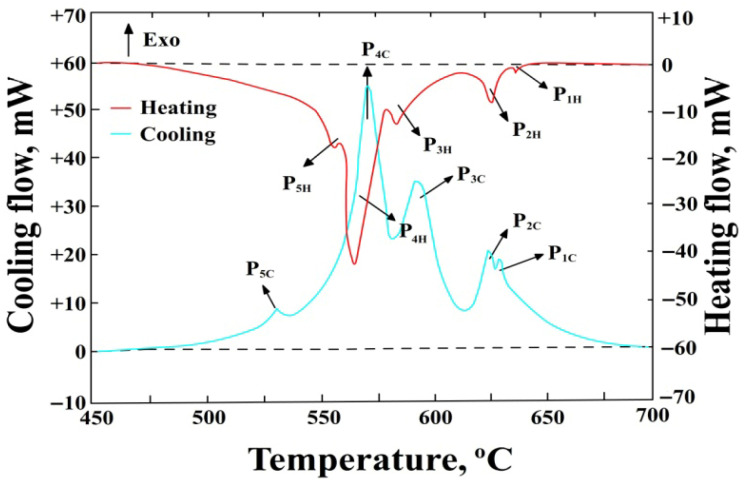
DSC curve of AlSi7Mg alloy during heating and cooling (alloy number 1).

**Figure 3:**
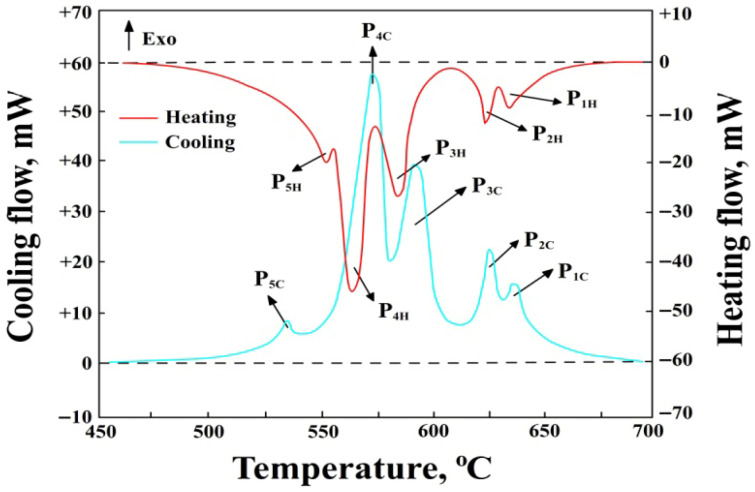
DSC curve of AlSi7Mg alloy during heating and cooling (alloy number 12).

DSC tests were performed for each of the four iron contents at the minimum and maximum manganese content, i.e., for alloy numbers: 1, 4, 5, 8, 9, 12, 13, and 16. A typical heating and cooling curve obtained for AlSi7Mg alloy number 1 (1.01 wt.% Fe + 0.11 wt.% Mn) is shown in Figure 2, while that for alloy no. 12 (1.4 wt.% Fe + 0.69 wt.% Mn) is shown in Figure 3.

The symbols in Figure 2 and Figure 3 denote:

- P_1H_—thermal effect from the precipitation of sludge particles during heating, P_1C_—during cooling, mW,
- P_2H_—thermal effect from the precipitation of α(Al) solid solution dendrites during heating, P_2C_—during cooling, mW,
- P_3H_—thermal effect from the precipitation of AlFeMn-type phases during heating, P_3C_—during cooling, mW,
- P_4H_—thermal effect from the precipitation of the double eutectic α(Al)+β(Si) during heating, P_4C_—during cooling, mW,
- P_5H_—thermal effect from the precipitation of the eutectic containing the Mg_2_Si phase during heating, P_5C_—during cooling, mW,
- Endo—endothermic reactions,
- Exo—exothermic reactions,
--------—baseline determining the area under the peak for calculating the enthalpy ΔH value.

A collective summary of the ranges of characteristic temperatures for the precipitation of structural constituents (during heating and cooling) for selected DSC tests is presented in Table 2.

Analysis of the DSC results allowed for the determination of the crystallisation sequence of the AlSi7Mg alloy. The primary phase to crystallise is sludge particles (from approx. 645 to 617 °C), with an increase in SF (Table 1) causing an increase in their precipitation temperature. Next, nucleation and crystallisation of α(Al) solid solution dendrites occur in the temperature range from approx. 628 to 617 °C-peaks P_2_. Their crystallisation temperature is consistent with the Al-Si equilibrium phase diagram for a content of approx. 7 wt.% Si [1]. After them, a multi-component eutectic, containing the AlFeMn-type phase, nucleates (from approx. 610 to 572 °C). An increase in the SF (Table 1) causes an increase in its crystallisation temperature, but it is always a crystallisation preceding the α(Al)+β(Si) eutectic. The next constituent of the AlSi7Mg alloy is the double eutectic α(Al)+β(Si), which crystallises from approx. 568 to 559 °C (peaks P_4_). The range of its crystallisation temperature is also consistent with the Al-Si equilibrium phase diagram and slightly lower than the temperature under equilibrium conditions (577 °C) [1,2]. Towards the end of its crystallisation, the Mg2Si phase nucleates and precipitates, being a constituent of the α(Al)+Mg_2_Si+β(Si) eutectic (from 554 to 540 °C), which is simultaneously the end of solidification of the AlSi7Mg alloy. Thus, the temperature ranges for the crystallisation of the α(Al) solid solution dendrites, the double α(Al)+β(Si) eutectic, and the triple α(Al)+Mg_2_Si+β(Si) eutectic are typical for the AlSi7Mg alloy, as confirmed in studies [43,44].

The primary reaction of sludge particles (before the formation of the α(Al) solid solution dendrites) deserves attention. From the DSC curves, it follows that as the SF value increases, the crystallisation of sludge particles not only occurs at a higher temperature but also the peak area associated with their precipitation becomes larger. This is related to the increasing value of heat released (during heating—an exothermic reaction) and absorbed (during cooling—an endothermic reaction) by the AlSi7Mg alloy. A similar regularity was observed for AlFeMn-type phases, meaning that an increase in the SF value is accompanied by an increase in crystallisation temperature (from 572 to 610 °C) and the area of the peaks associated with their precipitation. Therefore, measurements of enthalpy ΔH were performed during heating (+) and cooling (−), and a collective summary of these results is presented in Table 3.

To identify the phases whose reactions were recorded on the DSC curves, microstructure examinations of the AlSi7Mg alloy were performed.

### 3.3. Microstructure Test Results

**Figure 4:**
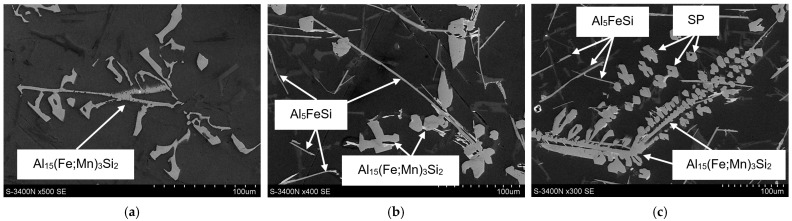
SEM microstructure of the AlSi7Mg alloy: (**a**) alloy no. 1 (SF = 1.37%); (**b**) alloy no. 6 (SF = 1.98%); (**c**) alloy no. 10 (SF = 2.14%).

Representative microstructures (SEM) of the AlSi7Mg alloy after the introduction of iron, manganese, and chromium are shown in Figure 4.

**Figure 5:**
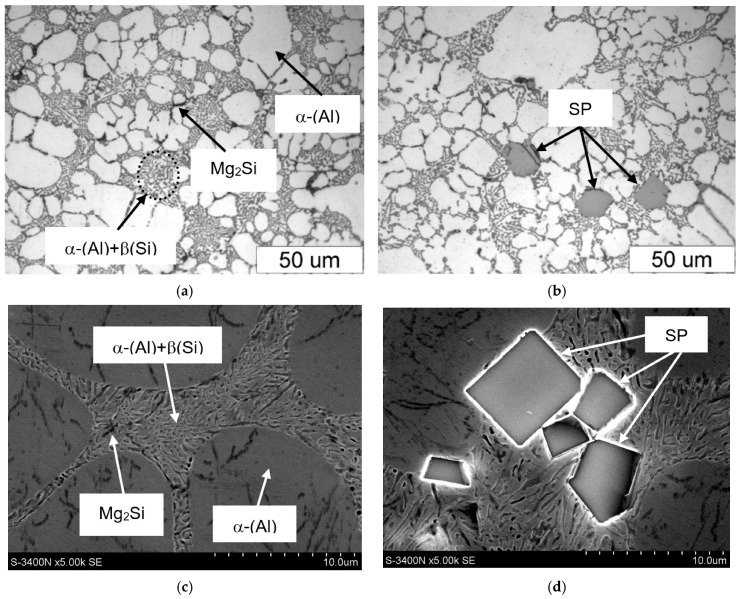
Microstructure of the AlSi7Mg alloy: (**a**,**c**) alloy no. 1 (without sludge particles); (**b**,**d**) alloy no. 11 (presence of sludge particles); (**a**,**b**) LM; (**c**,**d**) SEM.

**Figure 6:**
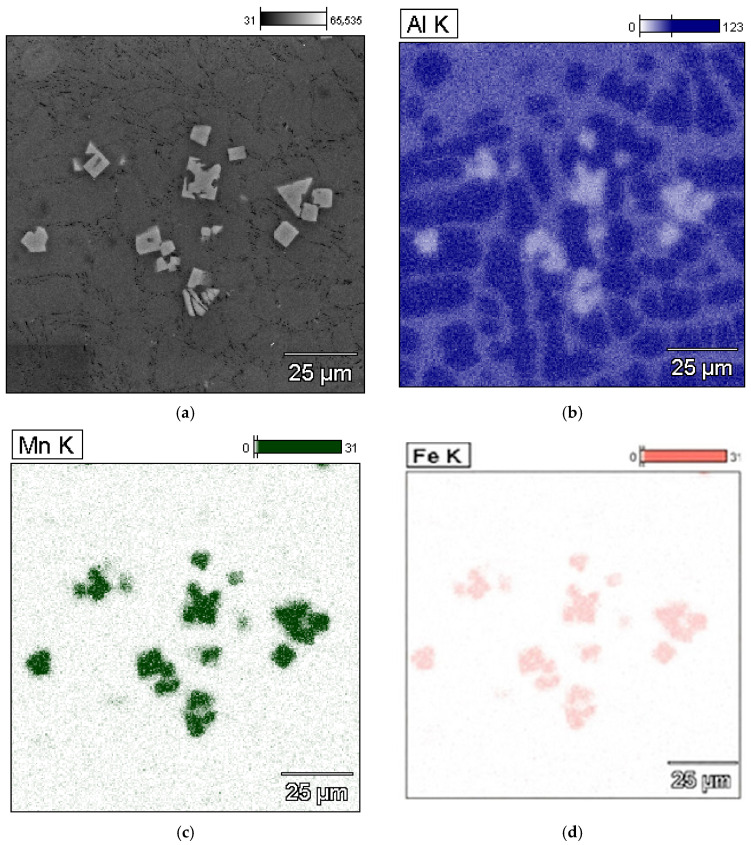
Microstructure (SEM) of the AlSi7Mg alloy containing sludge particles (**a**) and surface distribution of elements: (**b**) aluminium; (**c**) manganese; (**d**) iron.

From the microstructure identification and the authors’ own research [44], it follows that in the AlSi7Mg alloy, besides structural constituents—such as α(Al) dendrites; β(Si) crystals (part of the α(Al)+β(Si) eutectic); and the Mg_2_Si phase—part of the α(Al)+Mg_2_Si+β(Si) eutectic, the α-Al_15_(Fe;Mn)_3_Si_2_ phase, scarce precipitates of the β-Al_5_FeSi (β-Fe) and α-Al_13_(Fe;Cr)_4_Si_4_ [45] phases, and sludge particles were identified. From the microstructures presented in Figure 4, it follows that if SF < 1.5%, the microstructure of the AlSi7Mg alloy with an elevated share of iron and manganese is correct, and the (β-Fe) phases have been replaced by α-Al_15_(Fe;Mn)_3_Si_2_ with a typical dendritic structure. Increasing the SF from 1.5 to approx. 2.0% causes the appearance of scarce β-Fe precipitates, and the arms of the α-Al_15_(Fe;Mn)_3_Si_2_ phase become “thicker” (Figure 4b). If the sludge factor exceeds approx. 2.0%, then in the structure of the tested alloy, besides β-Fe and α-Al_15_(Fe;Mn)_3_Si_2_ phase precipitates, individual, “blocky” precipitates of sludge particles appear (Figure 4c). The microstructure of the AlSi7Mg alloy without sludge particles and with sludge particles is shown in Figure 5, and the surface distribution of aluminium, manganese, and iron in the sludge particles is shown in Figure 6.

**Figure 7:**
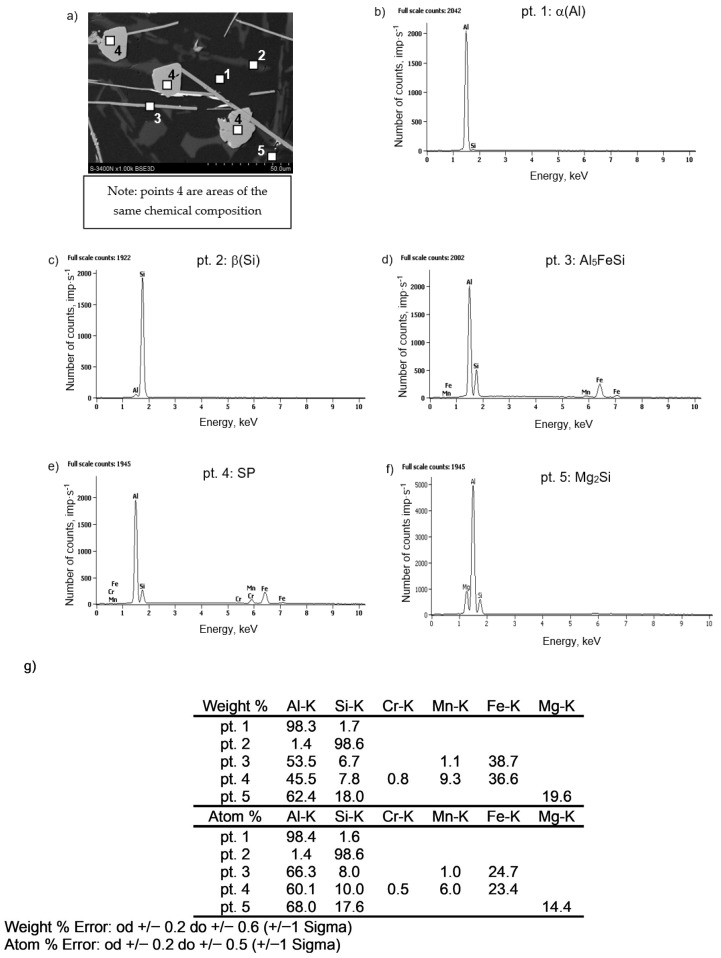
Microstructure (SEM) of the AlSi7Mg alloy containing sludge particles (**a**) and results of microanalysis of the chemical composition: (**b**) α(Al) dendrites, (**c**) β(Si) crystals, (**d**) Al_5_FeSi phase, (**e**) sludge particles, (**f**) Mg_2_Si phase, (**g**) element content at the investigated points.

The microstructure containing sludge particles and the results of microanalysis of the chemical composition of the AlSi7Mg alloy are presented in Figure 7.

**Figure 8:**
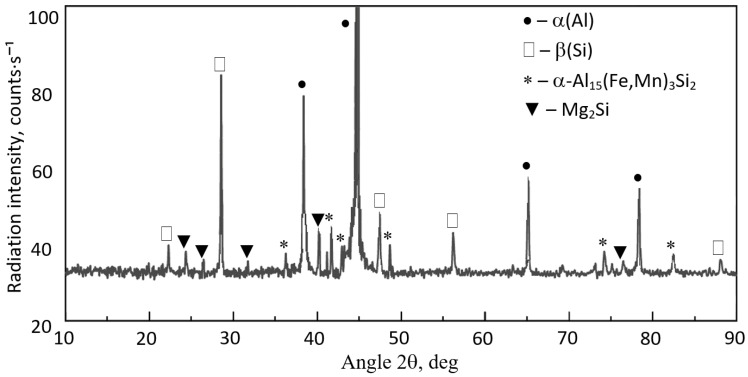
XRD diffraction pattern of AlSi7Mg alloy with manganese addition (sample no. 3).

To identify the structural constituents and intermetallic phases revealed in the microstructure of the AlSi7Mg alloy after introducing the Al-Mn master alloy, XRD investigations were carried out. The X-ray diffraction results are presented in Figure 8.

**Figure 9:**
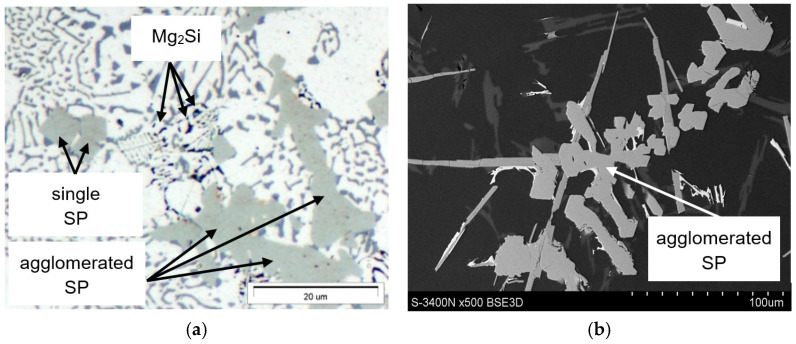
Microstructure (**a**) LM and (**b**) SEM of the AlSi7Mg alloy showing the varying morphology of sludge particles: (**a**) magnification 1000×; (**b**) magnification 500×.

Based on microscopic examinations, it can be stated that the structure of the AlSi7Mg alloy is correct. It corresponds to hypoeutectic Al-Si-Mg alloys with an increased content of iron and manganese. These elements (along with chromium) form sludge particles. Their morphology (similar to the Al_15_(Fe;Mn)_3_Si_2_ phase) changes with an increase in the SF value. Individual polyhedra “agglomerate” to form more massive clusters, as shown in Figure 9. However, explaining this phenomenon requires additional research.

It should therefore be noted that an increase in iron and manganese content affects not only the change in morphology of the Al_15_(Fe;Mn)_3_Si_2_ phase (from typically dendritic to more “fluffy”) but also the change in the structure of sludge particles from individual polyhedra (appearing as polygons on the polished surface) to compact “cluster-like” structures.

## 4. Discussion

The research aimed to identify phase transformations during the heating and cooling of an AlSi7Mg alloy with increased iron and manganese content using Differential Scanning Calorimetry (DSC) and to characterise the constituents of the alloy’s microstructure, with particular emphasis on so-called sludge particles (SP).

As research suggests [46], identifying phase transformations related to the crystallisation of intermetallic phases containing iron and manganese is difficult due to the influence of many factors, particularly the cooling rate and chemical composition. The method best suited for this is Differential Scanning Calorimetry (DSC).

From the DSC studies, it follows that the crystallisation of the AlSi7Mg alloy with increased iron and manganese content (at constant chromium content) proceeds according to the following sequence:

Liq. → SP + α(Al) + Al_15_(Fe,Mn)_3_Si_2_ + [α(Al) + β(Si)]^E^ + [α(Al) + (Mg_2_Si) + β(Si)]^E^

where the eutectics (^E^) may crystallise simultaneously.

The DSC investigation revealed that the crystallisation of the α(Al) dendrites occurs within the temperature range of approximately 618 °C to 626 °C (Table 2). Research [46] found that this range is between 588 and 593 °C in an AlSi9Cu3(Fe) alloy without manganese and between 594 and 604 °C with manganese. However, it is worth noting that the studied alloy had a higher silicon content (9 wt.%), which is consistent with the Al-Si equilibrium phase diagram. Furthermore, the AlSi9Cu3 alloy contains between 0.03 and 2.30 wt.% Cu, and as indicated by studies [46], copper lowers the crystallisation temperature of the α(Al) dendrites.

The temperature ranges for the crystallisation of AlFeMn-type phases and the α(Al)+β(Si) eutectic are also consistent with the results of the study [46]. The authors of the manuscript [46] also note that slight differences in temperatures for individual DSC measurements (up to approx. 15 °C) may occur, resulting from the cooling rate and chemical composition. As evidenced by research [47], the accurate analysis of phase transformations is most precise when samples are heated and cooled slowly at rates up to 20 °C min^−1^ (as was performed in the present manuscript). Higher rates can cause thermal effects to “overlap,” leading to misinterpretation of the DSC results.

The enthalpy during the formation of AlFeMn-type phases ranges from approx. 18 to 30 J·g^−1^ (Table 3) and is similar to the results of study [48], although potential differences in ΔH values may stem from varying iron and manganese contents and differences in the stoichiometry of these phases. The type of heat treatment applied is also significant. The results of the study [49] indicate that despite similar alloys (AlSi10Mg0.3Mn and 319), differences in ΔH values can amount to several J·g^−1^. Table 3 also shows that the enthalpy of the sludge particles ranges from approx. 5 to 12 J·g^−1^. Although these particles have been identified in numerous different works [36,41], the corresponding ΔH values are not provided therein, making it difficult to relate the obtained results to other studies.

The temperature ranges of crystallisation of the main structural constituents of the tested alloy are consistent with the results of works [1,2] and the sequence of crystallising phases as observed in studies [5,8]. The addition of manganese causes the transformation of plate-like/needle-like precipitates of the β-Al_5_FeSi phase into the α-Al_15_(Fe,Mn)_3_Si_2_ phase, characterised by a dendritic morphology (sometimes referred to as “Chinese script”). The presence of the α(Al)+Mg_2_Si+β(Si) eutectic containing the Mg_2_Si phase was confirmed. The research indicates that this eutectic forms at the end of the solidification of the AlSi7Mg alloy (approx. 554 to 540 °C). In Al-Si-Mg alloys with high iron and manganese content, it is not a constituent of sludge particles (Figure 7) and does not influence their morphology. This is confirmed by the results of studies [13,28].

Microstructure examinations of the AlSi7Mg alloy with elevated iron and manganese content also revealed the presence of sludge particles (SP). It was found that if the sludge factor [6] is up to approx. 1.5% (for high-pressure die-casting), the plate-like precipitates of the β-Fe phases are replaced by α-Al_15_(Fe;Mn)_3_Si_2_, which has a typical dendritic structure. Increasing the sludge factor from 1.5 to approx. 2.0% causes the appearance of scarce β-Fe precipitates (with lengths from 150 to 250 μm), and the arms of the Al_15_(Fe;Mn)_3_Si_2_ phase become thicker (Figure 4b,c). No additional AlFeMn-type phases were found in the structure of the AlSi7Mg alloy, only the rarely occurring Al_13_(Fe;Cr)_4_Si_4_ phase. When the sludge factor exceeds approx. 2.0%, in the structure of the tested alloy, besides β-Fe and Al_15_(Fe;Mn)_3_Si_2_ phases, sludge particles appear in the form of individual polygons (Figure 5d and Figure 7). Increasing iron and manganese content (SF > 2.7%) causes the arms of the Al_15_(Fe;Mn)_3_Si_2_ phase to become even “thicker,” and the sludge particles (rich in these elements) “agglomerate,” forming more massive clusters (Figure 8). This is probably because the “excess” iron and manganese atoms, which were not “bound” in the form of the Al_15_(Fe;Mn)_3_Si_2_ phase, formed the primarily crystallising sludge particles, settling at the bottom of the crucible (ladle), reducing the yield of the casting process. These observations are consistent with the results of studies [6,36]. Furthermore, it was found that the increase in the crystallisation temperature of sludge particles is directly proportional to the increase in the value of the sludge factor: from 620 °C (for SF~1.3%) to approx. 645 °C (for SF~3.1%) (Table 1 and Table 2). Hence, it can be stated that the crystallisation temperature of particles constituting the sediment sludge depends on the SF value, and therefore on the combined content of iron, manganese, and chromium. This is consistent with the sludge factor equation [33,36] and the results of studies [6,32], which present so-called “segregation curves” indicating critical values for preventing sediment formation.

Despite numerous studies on sludge particles [36,41], the literature lacks a quantitative assessment of the morphology of these particles. An exception is the research [6] concerning the AlSi9Cu3(Fe) alloy. It shows that an increase in iron and manganese content causes a linear increase in the average surface area and number of sludge particles. At the same time, the addition of chromium has no effect. The microstructure results of the AlSi7Mg alloy (Figure 4 and Figure 8) confirm these trends, although a quantitative analysis of the distribution of these particles was not performed. This will be the subject of further research on the microstructure of sludge particles in Al-Si alloys obtained from secondary materials.

Furthermore, the authors of the study [6] indicate that to better understand the morphology and distribution of sludge particles in Al-Si alloys, FEG-SEM studies after deep etching are necessary. This would allow for better revelation of the interior of both AlFeMn-type phases and sludge particles, which exhibit a structure of rhombic dodecahedra with empty spaces inside [6].

Although attempts to identify factors influencing the morphology of sludge particles can be found, for example, in studies [36,41], many issues regarding the crystallisation mode of these particles remain unclear. This concerns, for example, their nucleation mechanism [5,19], type of technology [25,41], cooling rate [4,33], and influence on the service properties of aluminium alloys produced from secondary components. These discrepancies underscore the need for further research in this area.

## 5. Conclusions

The following conclusions have been formulated based on the research conducted:

1. DSC tests revealed the primary crystallisation of sludge particles (SP), which, due to their weight, settle at the bottom of the crucible (ladle), reducing the casting process yield and are essential in controlling the microstructure of the AlSi7Mg(Fe) alloy.
2. The increase in the crystallisation temperature of SP, rich mainly in iron, manganese, and chromium, is directly proportional to the increase in the value of the sludge factor (SF) and ranges from 620 °C (for SF~1.3%) to approx. 645 °C (for SF~3.1%).
3. The combined increase in iron and manganese content influences not only the increase in the precipitation temperature of SP but also the change in their morphology from individual polyhedra (appearing as polygons on the polished surface) to compact “cluster-like” structures.
4. To avoid the formation of sludge particles with an unfavourable structure in high-pressure die-castings made of AlSi7Mg alloy, the SF should not exceed 2.0%.
5. DSC tests revealed the pre-eutectic crystallisation of the α-Al_15_(Fe;Mn)_3_Si_2_ phase, whose morphology depends on the combined content of iron, manganese, and chromium. For SF < 1.5%, the microstructure of the AlSi7Mg alloy with an elevated share of iron and manganese contains the α-Al_15_(Fe;Mn)_3_Si_2_ phase with a typical dendritic structure. A higher SF value leads to a significant thickening of the arms of the α phase.
6. The Mg_2_Si phase identified in the AlSi7Mg alloy (crystallising last) is not a constituent of sludge particles and does not influence their morphology.

## Figures and Tables

**Table 1:** Results of the chemical composition of the AlSi7Mg alloy.

Alloy No.	Element Content, wt.% ^1; 2^										Quotient Mn/Fe	Quotient Cr/Fe	SF
	Si	Fe	Cu	Mg	Ni	Mn	Zn	Ti	Cr	Sr			
1.	6.91	1.01	0.05	0.59	0.02	0.11	0.03	0.12	0.048	0.014	0.109	0.048	1.374
2.	7.11	0.91	0.02	0.52	0.03	0.31	0.01	0.12	0.048	0.014	0.341	0.053	1.674
3.	6.87	1.00	0.02	0.57	0.02	0.48	0.02	0.16	0.048	0.013	0.480	0.048	2.104
4.	6.94	0.96	0.07	0.52	0.01	0.69	0.04	0.16	0.047	0.014	0.719	0.049	2.481
5.	7.06	1.21	0.05	0.54	0.04	0.09	0.01	0.15	0.050	0.013	0.074	0.041	1.540
6.	7.07	1.22	0.07	0.55	0.05	0.31	0.01	0.11	0.049	0.015	0.254	0.040	1.987
7.	6.94	1.20	0.01	0.51	0.03	0.51	0.01	0.16	0.050	0.014	0.425	0.042	2.370
8.	7.01	1.19	0.01	0.60	0.01	0.71	0.02	0.13	0.050	0.013	0.597	0.042	2.760
9.	6.95	1.39	0.06	0.63	0.04	0.11	0.02	0.09	0.051	0.014	0.079	0.037	1.763
10.	6.87	1.39	0.08	0.61	0.05	0.30	0.04	0.11	0.051	0.011	0.216	0.037	2.143
11.	7.06	1.41	0.02	0.61	0.03	0.56	0.03	0.10	0.047	0.012	0.397	0.033	2.671
12.	6.88	1.40	0.04	0.59	0.02	0.69	0.02	0.13	0.050	0.014	0.493	0.036	2.930
13.	6.90	1.59	0.04	0.60	0.01	0.10	0.02	0.09	0.049	0.014	0.063	0.031	1.937
14.	7.02	1.59	0.03	0.58	0.03	0.31	0.04	0.12	0.049	0.015	0.195	0.031	2.357
15.	6.98	1.61	0.06	0.59	0.02	0.49	0.03	0.15	0.050	0.013	0.304	0.031	2.740
16.	7.03	1.60	0.02	0.55	0.01	0.71	0.02	0.14	0.049	0.014	0.444	0.031	3.167

^1^—remainder aluminium, ^2^—impurities, e.g., lead; tin—separately up to 0.05, together up to 0.02 wt.%.

**Table 2:** Results of DSC tests during heating and cooling of the AlSi7Mg alloy for selected experiments.

Alloy No.	Heating						Cooling				
	T, °C	E(Mg)	α+β	AlFeMn	α(Al)	SP	SP	α(Al)	AlFeMn	α+β	E(Mg)
1.	T_start_	542	559	572	618	621	620	620	572	561	548
	T_end_	553	568	596	628	631	630	625	592	568	552
4.	T_start_	540	560	574	617	621	620	619	575	560	541
	T_end_	552	565	597	627	635	633	622	595	568	554
5.	T_start_	541	559	577	619	625	622	618	577	559	540
	T_end_	554	562	597	625	634	635	624	599	567	550
8.	T_start_	540	561	577	618	626	626	620	577	560	547
	T_end_	552	565	600	624	637	638	623	600	565	549
9.	T_start_	539	558	577	618	625	626	619	577	561	541
	T_end_	550	564	602	626	640	640	626	602	564	554
12.	T_start_	543	560	578	619	623	626	621	577	560	547
	T_end_	554	568	604	626	641	641	626	605	565	552
13.	T_start_	544	561	580	619	626	626	620	580	559	541
	T_end_	552	567	606	624	643	643	625	608	564	554
16.	T_start_	540	563	580	618	627	626	618	580	560	546
	T_end_	550	566	607	625	645	645	624	610	566	550

Where T_start_—temperature of the start of the chemical reaction (transformation), T_end_—temperature of the end of the chemical reaction (transformation), α(Al)—aluminium solid solution dendrites, α+β—double eutectic α(Al)+β(Si), E(Mg)—eutectic containing the Mg_2_Si phase, AlMnFe—phases rich mainly in manganese and iron, SP—sediment sludge particles.

**Table 3:** Value of enthalpy ΔH of precipitation of sludge particles and AlFeMn-type phases during heating and cooling of the AlSi7Mg alloy for selected experiments.

Alloy No.	Component	ΔH, J·g^−1^	
		Heating	Cooling
1.	SP	+5	−6
	AlMnFe	+19	−18
4.	SP	+6	−6
	AlMnFe	+22	−20
5.	SP	+7	−6
	AlMnFe	+24	−23
8.	SP	+8	−7
	AlMnFe	+27	−27
9.	SP	+8	−7
	AlMnFe	+29	−28
12.	SP	+9	−9
	AlMnFe	+29	−30
13.	SP	+10	−10
	AlMnFe	+30	−30
16.	SP	+12	−12
	AlMnFe	+30	−30

## Data Availability

The original contributions presented in this study are included in the article. Further inquiries can be directed to the corresponding author.
